# Effects of Short-Term Annealing on the Thermal Stability and Microstructural Evolution of Oxygen-Free Copper Processed by High-Pressure Torsion

**DOI:** 10.3390/ma17235886

**Published:** 2024-12-01

**Authors:** Meshal Y. Alawadhi, Abdulkareem S. Aloraier, Ayman M. Alaskari, Abdullah A. Alazemi, Yi Huang

**Affiliations:** 1 Department of Manufacturing Engineering Technology, The Public Authority for Applied Education and Training (PAAET), College of Technological Studies, Shuwaikh 70654, Kuwait; as.aloraier@paaet.edu.kw (A.S.A.); am.alaskari@paaet.edu.kw (A.M.A.); 2 Mechanical Engineering Department, College of Engineering Petroleum, Kuwait University, P.O. Box 5969, Safat 13060, Kuwait; a.alazemi@ku.edu.kw; 3 Department of Design and Engineering, Faculty of Science and Technology, Bournemouth University, Poole, Dorset BH12 5BB, UK; yhuang2@bournemouth.ac.uk

**Keywords:** short-term annealing, thermal stability, dynamic recovery, softening, recrystallization, high-pressure torsion, HPT

## Abstract

This study explores the impact of short-term annealing on the thermal stability and mechanical properties of oxygen-free copper subjected to high-pressure torsion (HPT). Copper samples were deformed through HPT with varying numbers of turns at room temperature and subsequently subjected to short-term annealing at temperatures of 398 K and 423 K. Microstructural analysis revealed that annealing led to grain growth and a reduction in dislocation density, with samples processed with fewer HPT turns exhibiting more significant grain coarsening. The microhardness measurements indicated a reduction in hardness after annealing, particularly at the edges of the discs, suggesting recrystallization. Samples processed with 10 HPT turns demonstrated higher thermal stability and less grain growth compared to 1/2-turn samples. The findings suggest that post-HPT short-term annealing can be used to tailor the balance between strength and ductility in oxygen-free copper, enhancing its suitability for industrial applications.

## 1. Introduction

The key objective in numerous industrial applications is to use materials with enhanced strength and ductility. Grain size is a key factor in polycrystalline materials, as the Hall–Petch relationship suggests that reducing grain size leads to increased strength [1,2,3]. Furthermore, materials with a stable, fine grain structure tend to exhibit outstanding superplasticity at high temperatures, outperforming their coarser-grained equals [4,5,6,7].

Severe plastic deformation (SPD) is achieved by employing extremely high shear strain to bulk materials and has proven to be a highly effective method for producing ultrafine-grained (UFG) materials. This excellent grain refinement process is made possible by using significant hydrostatic pressure in combination with tools that have specialized geometries, which inhibit the free flow of material and maintain the shape of the specimen. Consequently, bulk UFG materials are produced without altering the overall dimensions [4,8]. While conventional thermomechanical processing limits grain refinement to a few micrometers, SPD can generate materials with an average grain size of less than 1 µm.

UFG materials with grain sizes below 100 nm are classified as nanocrystalline materials. Traditional processes like rolling, drawing, and extrusion encounter limitations during the application of high strain due to reduced cross-sectional dimensions and poor workability at room temperature. To overcome these challenges, SPD techniques have been developed to introduce high strain at low temperatures. These methods include high-pressure torsion (HPT), cyclic extrusion and compression (CEC), equal-channel angular pressing (ECAP), multi-directional forging (MDF), twist extrusion (TE), and accumulative roll bonding (ARB). Among these, HPT and ECAP are the most prominent for generating UFG materials. Studies have shown that HPT is the most efficient at achieving extremely fine grain sizes due to the accumulated strain during processing [9,10,11], whereas ECAP is favored for industrial applications as it can produce larger samples [12,13,14,15].

UFG materials are bulk materials distinguished by a uniform microstructure with equiaxed grains and grain boundaries displaying high-angle misorientation [16]. The intense shear strain introduced during SPD generates a high dislocation density within the bulk material. As these dislocations move and rearrange, a network of grain boundaries is formed.

The softening of copper can occur after HPT under certain conditions. The likelihood of softening copper after HPT is influenced by various factors, including the specific HPT processing parameters, temperature, strain rate, extent of deformation, initial microstructure, and impurity content. Dynamic recovery and dynamic recrystallization processes can take place, leading to a reduction in the overall hardness or strength of the material. By controlling the abovementioned factors, it is possible to minimize or avoid softening and achieve the desired copper microstructural and mechanical properties during HPT.

On the other hand, attaining structural stability of the materials produced by SPD is challenging, due to the potential usage in practical applications. Therefore, several studies have shown that proper annealing treatments are effective at improving the structural stability of UFG materials as well as enhancing their mechanical properties [17,18,19,20,21,22,23,24,25,26,27]. It has been shown that the strength of pure copper can be improved by subsequent annealing at 460 K for 10 min after twist extrusion [28]. Another study has shown that the strength enhancement of UFG Al, deformed by multiaxial compression, can be achieved through an annealing treatment at 330 K for 30 min [29]. Also, an improvement in strength and plasticity in pure magnesium has been reported when a post-annealing at 420 K, after hydrostatic extrusion, was applied [30]. An elongation of 39% and a UTS of 750 MPa have been recorded after annealing vanadium at 873 K for 15 min post HPT [31]. Strength and ductility of the ZK60 magnesium alloy have been successfully enhanced through annealing at 473 K for 20 min [32]. Recently, the Al-30Zn alloy has been processed by SPD and then heat treated through short-term annealing, with the results revealing an increase in the elongation of up to 40%, although the yield stress decreased [33]. A later study has reported that the application of short-term annealing at 573 K for 15 min on pure Ti after ECAP results in high strength and good ductility [34].

The aim of this study is to investigate the significance of the short-term annealing on the thermal stability of UFG oxygen-free copper. This study examines the effect of microstructural evolution on the strength and ductility of UFG structure as a function of the annealing temperature. 

## 2. Materials and Methods

**Figure 1:**
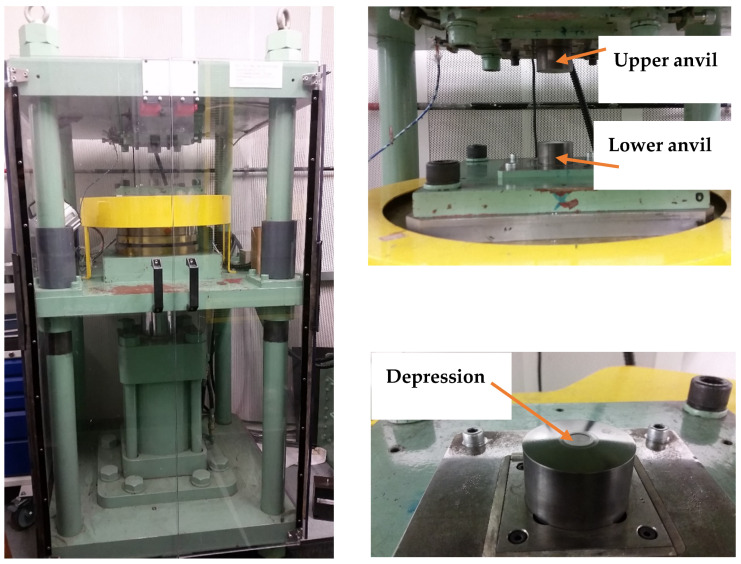
High-pressure torsion setup.

The experiment was performed using commercial C10300 oxygen-free copper with a nominal composition of 99.95% Cu and 0.003% P. The material was initially cut into discs, each with a diameter of 10 mm and a thickness of ~0.85 mm, and then annealed for an hour at a temperature of 600 °C using a vacuum tube furnace to prevent oxygen contamination during the heat treatment process. After that, the discs were processed by HPT at room temperature under quasi-constrained conditions [35], allowing for limited material outflow between the anvils. The HPT setup shown in Figure 1 consists of upper and lower anvils, both featuring a circular depression of 10 mm in diameter and 0.25 mm in depth [36]. The disc was placed in the depression of the lower anvil, which was then moved upward to compress the disc under an applied pressure of 6.0 GPa. Torsional strain was applied by rotating the lower anvil at 1 rpm, with the pressure maintained throughout. Copper discs were processed at room temperature (298 K) with two numbers of HPT turns, including N = 1/2, and 10. After processing each disc, both anvils were thoroughly cleaned with ethanol to ensure a contamination-free process.

The microstructural properties of oxygen-free copper were examined both in the annealed state and after processing through HPT. All specimens were ground using silicon carbide (SiC) paper up to 4000 grit, followed by polishing with 9 µm, 6 µm, 3 µm, and 1 µm diamond suspensions, and, finally, polished with a vibratory machine using a 0.4 µm colloidal silica solution. Microstructural data were acquired with a JEOL JSM-7001 F analytical field emission scanning electron microscope (FE-SEM) at an operating voltage of 15 kV. Data collection was performed using electron backscatter diffraction (EBSD) and orientation imaging microscopy (OIM™), using the Aztec, Oxford OIM software version 4.0 and a TSL orientation imaging system. EBSD patterns were captured at a working distance of 15 mm with a 70° sample tilt. OIM images were generated with a step size of 0.05 µm. In the analysis, high-angle grain boundaries were defined by a misorientation angle of >15° between adjacent measurement points, while low-angle grain boundaries (LAGBs) were classified by a misorientation angle ranging from 2° to 15°.

The X-ray specimens were ground and polished following the same metallographic methods used for optical microscopy. To eradicate the deformed layer on the surface of the specimens, they were lightly etched with a solution containing a mixture of 50 mL distilled water and 50 mL nitric acid. The surfaces of the specimens were analyzed using a Bruker D2 Phaser X-ray diffractometer that is operational with a copper target and employing Cu Kα radiation (λ = 0.15406 nm). XRD patterns were obtained through θ–2θ scans, covering a 2θ range of 30° to 100°. Microstructural parameters, such as lattice parameter, crystallite size, and microstrain were measured using the materials analysis software (MAUD) version 2.9992, which is based on the Rietveld method [37,38,39], a comprehensive full-pattern fitting technique.

Microhardness measurements were performed using a Future-Tech Microhardness tester, FM-300, with an indentation load of 100 gf and a dwell time of 15 s. Average values were taken along the diameter of each disc, with measurements spaced 0.3 mm apart. Four indentations were made at each point, with a 0.15 mm separation between them, and the average of these four measurements was calculated. To prevent interference from plastic deformation zones surrounding each indentation, the spacing between indentations was maintained at three times the indentation size.

Tensile tests were conducted at room temperature using a tensile testing machine, and the samples were pulled to failure under an initial strain rate of 1.0 × 10^−3^ s^−1^. Two miniature tensile samples were cut from each HPT disc using a wire electrical discharge machine. The center of each tensile sample was 2 mm from the center of the disc to avoid the non-uniform microstructure in the central region.

## 3. Results

### 3.1. Microhardness

**Figure 2:**
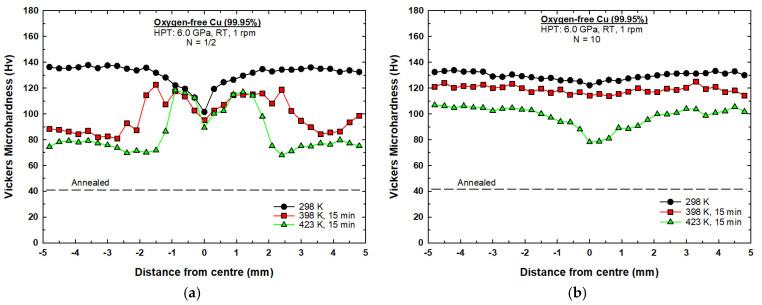
Hardness measurements along the diameter of the disc processed by HPT at 298 K and discs subjected to short-term annealing at temperatures of 398 K and 423 K for 15 min: (**a**) 1/2 turn; (**b**) 10 turns.

Figure 2a shows the hardness measurements along the diameter of the 1/2 turn disc and the 1/2 turn discs subjected to post-HPT short-term annealing for 15 min at temperatures of 398 K and 423 K. It is apparent from Figure 2a that the microhardness values of the 1/2 turn Cu sample were significantly higher compared to the hardness value of the material in initial annealing state (~41 Hv, shown by the lower dashed line). It can be seen that microhardness values deviated along the diameter of the disc processed by 1/2 turn. A lower average microhardness value of ~100 Hv was recorded at the center of the disc and increased toward the edges to a maximum average value of ~137 Hv.

The microhardness values dropped significantly after post-HPT annealing at 398 K for 15 min, as shown in Figure 2a. The hardness values varied across the diameter of the disc, with no evident change in the center of the disc, but the hardness values dropped gradually moving away from the disc center, up to a distance of ~2.0 mm. Beyond ~2.0 mm from the center of the disc, a substantial decline in the hardness values was observed, moving toward the peripherals where a minimum average value of ~85 Hv was recorded.

Increasing the post-HPT annealing temperature to 423 K showed a similar harness variation trend to the one observed at 398 K, as shown in Figure 2a. It is readily apparent that there was no change occurring in the central area of the disc (from 0 to ~1.0 mm). The significant softening in hardness started ~1.5 mm from the disc center, moving toward the peripherals where a minimum average value of ~75 Hv was recorded.

Figure 2b shows the hardness measurements along the diameter of the 10-turn disc and the 10-turn discs subjected to short-term annealing for 15 min at temperatures of 398 K and 423 K. The hardness values across the diameter of the 10-turn disc were reasonable. Under this condition, the values across the diameter of the disc were stable, with an average value of ~130 Hv, which suggests that some dynamic recovery occurred during the HPT process.

Minor softening occurred after post-HPT annealing at a temperature of 398 K for 15 min where the hardness values were also reasonably homogenous across the diameter of the disc, attaining an average value of ~120 Hv, as shown in Figure 2b. At 423 K, post-HPT annealing, the hardness values continued to decrease to an average value of ~105 Hv from ~1 mm to 5 mm from the center of the disc. Lower hardness values were observed in the central region of the disc between ~(−1 mm) and ~(1 mm), with the center of the disc showing the lowest value of ~80 Hv.

### 3.2. X-Ray Diffraction

**Figure 3:**
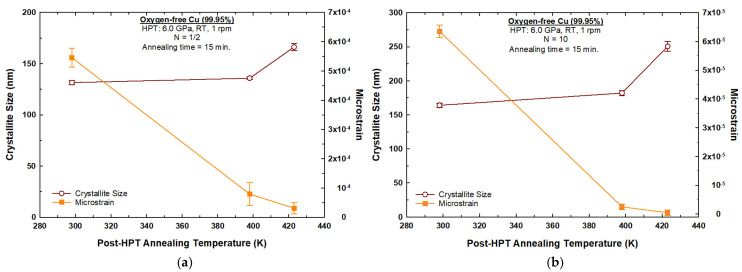
Crystallite size and microstrain with respect to the samples processed by HPT at 298 K and at post-HPT annealing temperatures for: (**a**) 1/2-turn sample; (**b**) 10-turn sample.

Figure 3 shows variations in the crystallite size and microstrain of the 1/2-turn and 10-turn samples with respect to the post-HPT annealing temperature. It is readily apparent from Figure 3a,b that microstrain decreased, while crystallite size increased, with increasing annealing temperatures. The XRD analysis on the 1/2-turn sample found a microstrain of ~5.45 × 10^−4^ and a crystallite size of ~131 nm. After post-HPT annealing at a temperature of 398 K for a period of 15 min, the microstrain value decreased to ~7.92 × 10^−5^, whereas the crystallite size increased to ~136 nm, as shown in Figure 3a. Increasing the post-HPT annealing temperature to 423 K led to a decrease in the microstrain value (~3.09 × 10^−5^) and an increase in the crystallite size (~166 nm).

On the other hand, the microstrain and crystallite size of the 10-turn sample were ~6.35 × 10^−5^ and 164 nm, respectively, as shown in Figure 3b. After post-HPT annealing at a temperature of 398 K for a period of 15 min, the microstrain value decreased to ~2.49 × 10^−6^, whereas the crystallite size increased to ~182 nm, as shown in Figure 3b. Increasing the annealing temperature to 423 K led to a decrease in the microstrain value (~5.0 × 10^−7^) and an increase in the crystallite size (~250 nm).

**Figure 4:**
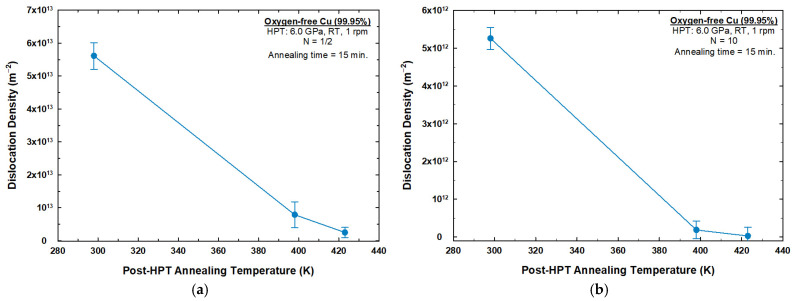
Dislocation density as a function of post-HPT annealing temperatures for: (**a**) 1/2-turn sample; (**b**) 10-turn sample.

Figure 4 shows the dislocation density as a function of post-HPT annealing temperatures for the 1/2-turn and 10-turn samples. The calculated dislocation densities for the 1/2-turn and 10-turn samples were ~5.61 × 10^13^ m^−2^ and ~5.26 × 10^12^ m^−2^, respectively. The values of dislocation densities decreased to ~7.90 × 10^12^ m^−2^ and ~1.85 × 10^11^ m^−2^ after post-HPT annealing at 398 K for the 1/2-turn and 10-turn samples, respectively. The dislocation densities continued to decrease to values of ~2.52 × 10^12^ m^−2^ and ~2.71 × 10^10^ m^−2^ under a higher annealing temperature of 423 K for both 1/2-turn and 10-turn samples, respectively.

### 3.3. Microstructural Evolution

**Figure 5:**
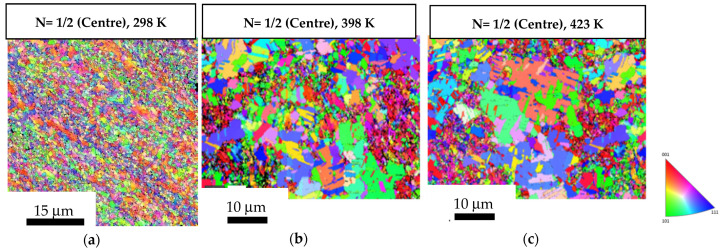
OIM images of the central regions of the 1/2-turn samples: (**a**) in a HPT-processed state; (**b**,**c**) subjected to short-term annealing at 398 K and 423 K.

**Figure 6:**
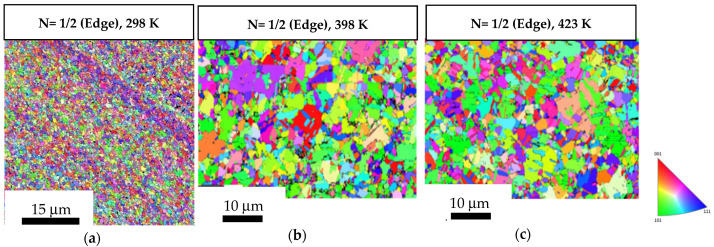
OIM images of the peripheral regions of the 1/2-turn samples: (**a**) in a HPT-processed state; (**b**,**c**) subjected to short-term annealing at 398 K and 423 K.

Figure 5 shows the OIM images of the central regions of the 1/2-turn discs without undergoing post-HPT annealing and after undergoing post-HPT short-term annealing at 398 K and 423 K. The measured average grain size in the central region of 1/2-turn discs was ~0.82 µm, as shown in Figure 5a. The average grain size increased to ~1.48 µm and ~1.57 µm after short-term annealing at 398 K and 423 K, respectively, as shown in Figure 5b,c. Figure 6 demonstrates a similar trend in the peripheral regions of the 1/2-turn discs without undergoing post-HPT annealing and after undergoing post-HPT short-term annealing. The average grain size in the peripheral region of the 1/2-turn discs was ~0.69 µm, as shown in Figure 6a, whereas the grain size increased to ~1.85 µm and ~1.96 µm after short-term annealing at 398 K and 423 K, respectively, as shown in Figure 6b,c. The results are consistent with the reduction in hardness values shown in Figure 2a at the edges of the discs after short-term annealing at 398 K and 423 K.

**Figure 7:**
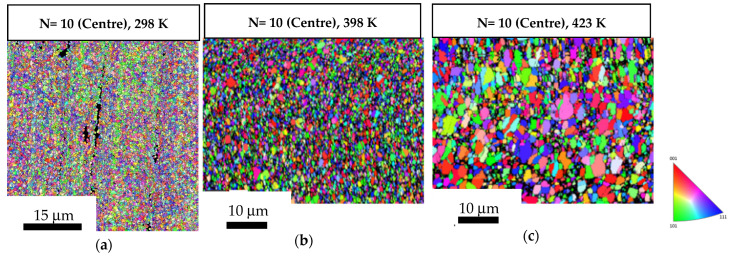
OIM images of the central regions of the 10-turn samples: (**a**) in a HPT-processed state; (**b**,**c**) subjected to short-term annealing at 398 K and 423 K.

**Figure 8:**
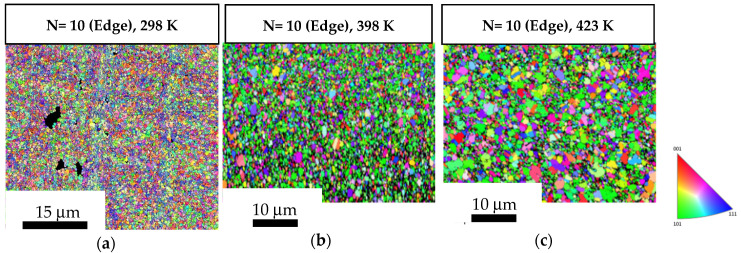
OIM images of the peripheral regions of the 10-turn samples: (**a**) in a HPT-processed state; (**b**,**c**) subjected to short-term annealing at 398 K and 423 K.

The average grain size was ~0.52 µm in both the central and the peripheral regions of the 10-turn samples, as shown in Figure 7a and Figure 8a, respectively. The central region of the discs showed average grain size values of ~0.89 µm and ~1.46 µm after post-HPT short-term annealing at 398 K and 423 K, as shown in Figure 7b,c whereas the peripheral regions showed average grain size values of ~0.96 µm and ~1.27 µm after short-term annealing at 398 K and 423 K, respectively (Figure 8b,c). Table 1 shows a summary of grain size values by comparing Figure 5 and Figure 6 with Figure 7 and Figure 8, also showing that the grain growth in the 10-turn samples was lower than that in the 1/2-turn samples. It is readily apparent that the discs subject to a higher number of turns had higher thermal stability after short-term annealing than the discs subject to a lower number of turns.

### 3.4. Tensile Properties

**Figure 9:**
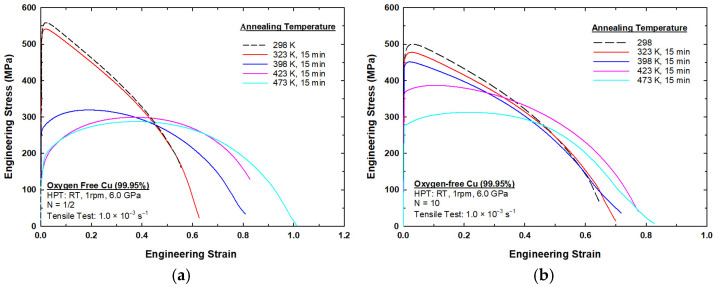
Stress–strain curves for samples processed by HPT at 298 K then short-term annealed for 15 min at various temperatures: (**a**) 1/2 turn; (**b**) 10 turns.

Figure 9 illustrates the relationship between engineering stress and engineering strain in relation to oxygen-free copper (99.95%) subjected to HPT at 298 K and to short-term annealing for 15 min at various temperatures: (a) 1/2-turn sample; (b) 10-turn sample. Figure 9a shows that the sample processed by HPT for 1/2 turn at 298 K exhibited a high UTS of ~560 MPa and a relatively low elongation to failure of ~55%. An inspection of the sample after post-HPT annealing at a temperature of 323 K showed that the UTS decreased slightly to ~540 MPa, while the elongation improved to around 63%, suggesting a slight increase in ductility. As the post-HPT annealing temperature rose to 398 K, the UTS dropped significantly to ~320 MPa, with elongation increasing to approximately 81%. Increasing the post-HPT annealing temperature to 423 K had a minor effect on the UTS and elongation values (~300 MPa and ~83%, respectively). A further increase in the post-HPT annealing temperature to 473 K showed a slight decrease in the UTS value to ~280 MPa, while the elongation to failure showed a substantial enhancement to approximately 110%.

Based on Figure 9b, the sample processed by HPT for 10 turns at 298 K displayed a UTS value of ~500 MPa, with an elongation to failure of ~65%. At a post-HPT annealing temperature of 323 K, the UTS decreased slightly to ~480 MPa, while the elongation improved to ~70%, suggesting a slight increase in ductility. At 398 K, the UTS further decreased to ~450 MPa, accompanied by a minor increase in elongation, reaching approximately 72%. Increasing the post-HPT annealing temperature to 423 K led to a decrease in the UTS to ~400 MPa and to an increase in the elongation to ~78%. Finally, at 473 K, the UTS decreased to ~300 MPa, while the elongation to failure increased to ~83%. The values of UTS and elongation to failure are recorded in Table 2, where the results illustrate that increasing the post-HPT annealing temperatures reduced the UTS while significantly enhancing elongation, reflecting the trade-off between strength and ductility in oxygen-free copper processed under these conditions.

## 4. Discussion

### 4.1. The Existence of Initial Softening During HPT Processing at 298 K

**Figure 10:**
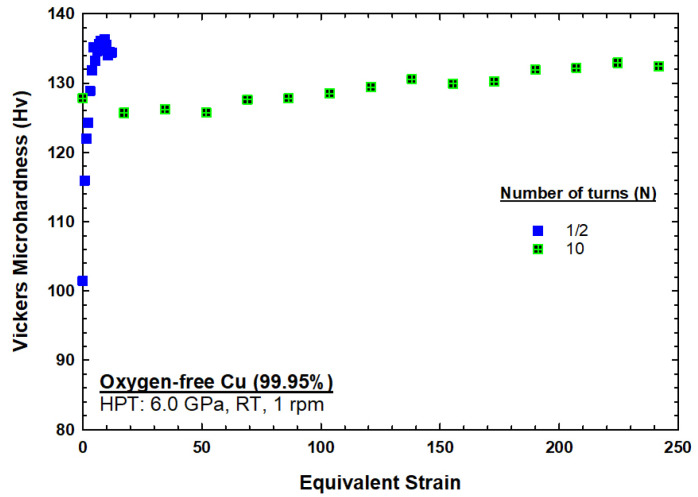
The variation in microhardness for the 1/2-turn and 10-turn discs at 298 K with respect to the equivalent strain.

Figure 2 shows the microhardness measurements recorded along the diameter of the discs processed by HPT for (a) 1/2 turn and (b) 10 turns. A similar graph has been previously reported for oxygen-free copper processed by HPT at 298 K for various numbers of turns [40]. In Figure 2a,b, it can be seen that the comparison of microhardness values recorded at 298 K showed that the microhardness values around the edges of the disc after 10 turns were lower than the microhardness values after 1/2 turn. The variation in microhardness in both curves at 298 K were plotted with respect to the equivalent strain, as shown in Figure 10. The results in Figure 10 show a transition from hardening to softening which suggests that dynamic recovery had taken place during the HPT processing. The results are in agreement with an earlier model that shows hardening followed by softening, with a rapid recovery based on the variations in microhardness values with respect to the equivalent strain [41,42,43]. A rapid increase in the hardness values was recorded in the initial stage of HPT straining, then a subsequent decrease occurred at an equivalent strain of ≈12, and then, after reaching an equivalent strain of ≈50, the hardness saturated at ~130 Hv, as shown in Figure 10. The results exactly match those obtained by an earlier study on oxygen-free copper (99.95 wt%) that reported the occurrence of dynamic recovery during the HPT process [40], while other studies on OFHC Cu (99.99+ wt%) processed by HPT at room temperature have reported that this phenomenon is associated with the occurrence of dynamic recrystallization during the HPT process [44,45]. The observed trend in Figure 10 is also consistent with earlier studies on high-purity copper (99.99 wt%); however, these studies show different values of hardness at saturation at different equivalent strain values [45,46,47]. A recent study has identified this behavior as three distinct stages which vary according to the equivalent strain values, and they consist of hardening, softening, and saturation [48]. During stage one, the dislocation density is accumulated at low equivalent strain. During stage two, microstructural inhomogeneity is observed at moderate equivalent strain. Stage three happens at higher equivalent strain values and corresponds to the stabilization of the microstructure, characterized by the presence of high-angle grain boundaries (HAGBs).

Another piece of evidence in support of the occurrence of dynamic recovery is the decrease in microstrain after 10 turns, shown in Figure 3b, compared to the microstrain value recorded after 1/2 turn, shown in Figure 3a at 298 K. Also, the dislocation value after 10 turns, shown in Figure 4b, was lower compared to the value obtained after 1/2 turn, shown in Figure 4a at 298 K. Temperature increases during the HPT processing is a factor that might influence the softening and act as a driving force for recovery; however, it has been shown in earlier studies that the temperature rise after 10 turns is minor and does not trigger recovery during the HPT process for oxygen-free copper [40].

### 4.2. Thermal Stability After Short-Term Annealing

A variation in microhardness values across the diameter of the copper disc was observed after 1/2 turn at 298 K, as shown in Figure 2a, where lower values were recorded in the central region and higher values were recorded with increasing distance from the center, reaching maximum values around the edges. The inhomogeneity in the microstructure was due to the variation in the equivalent strain across the disc (zero at the center and the maximum at the edge of the disc) based on the von Mises equation [48]:

(1) $$\varepsilon_{ev\;}=\frac{2\pi Nr}{h\sqrt{3}}$$

where *N* is the total number of turns, *r* is the radius of the disc, and *h* is the thickness (or height) of the disc. At higher strain values, the microhardness values reached a steady state and showed a uniform distribution across the diameter of the disc after 10 turns at 298 K, as shown in Figure 2b.

The results display an occurrence of significant softening around the edges of the disc processed through a lower number of turns (1/2 turn) after short-term annealing at 398 K and 423 K for 15 min, as shown in Figure 2a. It is believed that this softening may be attributable to the occurrence of recrystallization during the HPT process. This is justified by the variation in the imposed strain across the disc (von Mises) that led to higher friction between the edges of the disc and the anvil walls compared to the center. The higher friction generated additional dislocations that led to higher internal stored energy around the edges, providing the driving force for recrystallization during the short-term annealing. It is well documented that the dislocation density increases in UFG materials as the population of grain boundaries increases after SPD processing [49]. Also, UFG materials, having heterogenous microstructures, are more susceptible to recrystallization than UFG materials with homogenous microstructures due to the strain energy gradient [50,51]. An earlier report on pure Cu has shown the presence of finer grains with higher fractions of high-angle grain boundaries and twin boundaries in the edges of the disc compared to the center after HPT processing with a lower number of turns [40]. Places with HAGBs have been proved to be places where recrystallization is initiated in pure copper during annealing [52].

The variation in microhardness is related to the accumulation of strain across the disc induced by the deformation process. In the current study, the copper discs processed by HPT at 298 K showed a hardness gradient from the center to the edges due to the strain accumulated during HPT, where the strain is dictated by torsional mechanics, with strain highest at the edges. The center of the disc showed lower hardness due to the lower strain where the edges showed higher hardness because of higher strain. After the short-term annealing at 398 K and 423 K, the reduction in hardness was significant at the edges due to recrystallization and dynamic recovery, with some softening at the center. In the samples processed with fewer HPT turns (1/2 turn), grain coarsening further decreased hardness, especially at higher temperatures.

Other studies have shown a variation in hardness due to the variation of the imposed strain exerted on the material by other deformation processes. For example, during the radial shear rolling (RSR) of titanium alloy billets [53], the hardness in the radial direction of the billet has been shown to have a characteristic ring-shaped distribution, with hardness decreasing toward the core and attaining its maximum at the surface. This distribution is due to a greater accumulation of strain near the surface because of the rolling deformation. As a result, the hardness gradient following RSR is not as steep because of limited strain penetration. Another study has investigated ETP grade copper after a wire drawing process where the hardness of the wire was measured at two primary locations: the axis and the surface of the drawn wire [54]. Under non-stationary conditions, the hardness values along the axis were found to be significantly higher compared to those at the surface. This suggests that the material along the axis undergoes greater strain hardening due to a more concentrated deformation, thereby increasing hardness. Conversely, under stationary conditions, the hardness along the axis was found to be lower than that at the surface. This indicates that the distribution of the strain is more uniform across the surface, leading to a less localized hardening in the axis region.

It has been frequently recognized in the literature that, in HPT-processed materials, the hardness distribution is controlled mainly by the strain gradients at room temperature [40,55,56]. The contribution from impurity affects the recovery/recrystallization processes in relation to hardness where it is more pronounced at elevated temperatures, especially in the center [54]. The torsion strain applied by HPT dramatically decreases from the edge to center of a sample at room temperature. Shear deformation is much lower in the center than at the edges, and, because of this, dislocation density is also lower in the center, hence lowering strain hardening. While impurities (0.05%) can potentially increase hardness through strengthening mechanisms, they may also promote dynamic recovery in localized regions. The reduced strain at the center facilitates the occurrence of recovery processes, counteracting any hardening from impurity accumulation. As such, impurities in oxygen-free copper tend to accumulate along grain boundaries, especially during various deformation and thermal treatments. Impurity-driven strengthening effects could dominate at high temperatures, as it has been reported [54] in the context of wire drawing of ETP grade copper. However, the less deformed boundaries may not benefit so much from the segregation of impurities (at room temperature). Also, increasing processing or annealing temperatures improve impurity diffusion as well as recrystallization [53,54].

### 4.3. Strength and Ductility After Short-Term Annealing

By looking at the ultimate tensile stress (UTS) in both Figure 9a,b, it is obvious that the sample processed by HPT at 298 K exhibited the highest UTS. As the annealing temperature increased, the UTS progressively decreased in samples annealed at 323 K, 398 K, 423 K, and, finally, 473 K. This decrease in UTS was likely due to the reduction in dislocation density and softening caused by recovery and recrystallization during annealing.

On the other hand, the elongation to failure (ductility) corresponds to the strain at which the material fractures. As the annealing temperature increased, the elongation to failure increased, as shown in Figure 9a,b. The sample processed by HPT at 298 K had the lowest elongation, where the samples annealed at higher temperatures (e.g., 423 K and 473 K) showed significantly higher elongation, indicating increased ductility. This behavior can be attributed to the annealing process, which reduces material brittleness by relieving internal stresses and promoting grain growth.

A close inspection of Figure 9 reveals a general trend in relation to the short-term annealing temperatures. The samples exhibited high ultimate tensile strength (UTS) at low temperatures (298–323 K), but they also exhibited low elongation. This is a behavior typical of materials with high internal strain and high dislocation density. At intermediate temperatures (398 K), the samples attained a balance between strength and ductility, as recovery began to dominate. Samples tested at high temperatures (423–473 K) showed lower UTS and significantly higher elongation, both of which can be attributed to recrystallization and grain growth.

### 4.4. Softening Behaviour Similar to the Effect of 12-Month Storage

**Figure 11:**
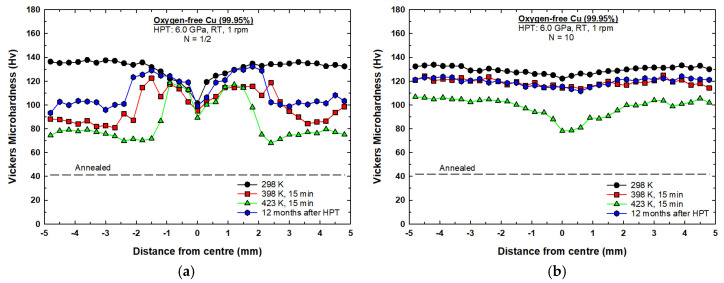
Comparison between the softening which occurred after the short-term annealing and after the long-term storage in relation to the samples processed by HPT: (**a**) 1/2 turn; (**b**) 10 turns.

Interestingly, the results observed in this study after the short-term annealing were similar to the results observed in copper discs after long-term storage at room temperature for 12 months [40]. It can be seen that temperature had a strong impact on the softening which occurred in the HPT-processed copper. Post-HPT annealing temperatures accelerated the softening process in the 1/2-turn disc compared to the softening which occurred in the 1/2-turn disc during storage at room temperature for 12 months. Also, the area exhibiting the softening effect, associated with an obvious decrease in hardness, extended more toward the center of the 1/2-turn disc sample post-HPT annealing at 423 K compared to the area affected by softening in the 1/2-turn disc sample subjected to post-HPT annealing at 398 K, as seen in Figure 11a. Furthermore, the same softening trend was observed in the 10-turn disc subjected to post-HPT short-term annealing at 398 K in comparison to the 10-turn disc subjected to storage at room temperature for 12 months, as shown in Figure 11b. Also, it can be seen that increasing the post-HPT annealing temperature to 423 K caused the central area of the 10-turn disc to experience a stronger softening effect than that documented in the disc edge area, as shown in Figure 11b.

## 5. Conclusions

In conclusion, this study provides valuable insights into the effect of short-term annealing on oxygen-free copper processed by high-pressure torsion (HPT). The research highlights the ability of short-term annealing to modify the microstructure of copper and achieve an optimal balance between strength and ductility. The variations in grain size, dislocation density, microstrain, and crystallite size after HPT and the subsequent annealing treatments demonstrate significant thermal stability improvements. The main findings of this study with respect to the effect of short-term annealing on oxygen-free copper processed by HPT are:

1. Microhardness variation:
  ○ The hardness of the copper discs increased significantly after HPT processing. The microhardness values varied across the diameter of the discs, with the center showing lower hardness values and the edges showing higher values due to the strain gradient.
  ○ Post-HPT short-term annealing at 398 K and 423 K caused a significant reduction in hardness, especially around the edges, indicating the occurrence of softening due to recrystallization.
2. Grain Size and Microstructural Evolution:
  ○ The grain size in HPT-processed samples increased with annealing temperatures. Samples processed with fewer HPT turns exhibited a more significant grain growth after annealing compared to those experiencing more turns.
  ○ Samples processed with 10 turns displayed greater thermal stability, retaining a finer grain structure after annealing compared to those processed with fewer turns.
3. Crystallite Size and Microstrain:
  ○ Crystallite size increased and microstrain decreased as the annealing temperature increased. This trend was consistent across both 1/2-turn and 10-turn samples, with the 10-turn sample showing less variation.
  ○ The dislocation density also decreased with increasing annealing temperatures, further confirming the occurrence of dynamic recovery and recrystallization.
4. Strength and Ductility:
  ○ A clear trade-off exists between ultimate tensile strength (UTS) and elongation. As the short-term annealing temperatures increased, oxygen-free copper samples became softer yet exhibited greater ductility.
  ○ This phenomenon illustrates the microstructural changes occurring in oxygen-free copper during the short-term annealing process, characterized by a reduction in dislocation density and an increase in grain size with rising temperatures.
5. Softening Mechanism:
  ○ The softening observed after short-term annealing was similar to that observed in samples subject to long-term storage at room temperature, suggesting that temperature accelerates the softening process.
  ○ The study found that areas with high-angle grain boundaries (HAGBs) were more prone to recrystallization during annealing, contributing to the softening of the material.

These findings have important implications for enhancing the mechanical properties of oxygen-free copper, making it more suitable for various industrial applications that require high performance under thermal and mechanical stress.

## Figures and Tables

**Table 1:** The average grain sizes of the central and peripheral regions of the 1/2-turn and 10-turn samples at 298 K (HPT-processed) and at 398 K and 423 K (subjected to post-HPT short-term annealing).

No. of Turns	Position	Grain Size (µm)		
		298 K	398 K	423 K
1/2 turn	Centre	0.82	1.30	1.21
	Edge	0.69	1.23	1.12
10 turns	Centre	0.52	0.37	0.54
	Edge	0.52	0.34	0.42

**Table 2:** UTS and elongation values recorded after conducting tensile testing on copper samples that were initially processed by HPT at 298 K then short-term annealed for 15 min at various temperatures.

	1/2 Turn		10 Turns	
Temp. (K)	UTS (MPa)	Elongation (%)	UTS (MPa)	Elongation (%)
298	560	55	500	65
323, 15 min.	540	63	480	70
398, 15 min.	320	81	450	72
423, 15 min.	300	83	400	78
473, 15 min.	280	110	300	83

## Data Availability

The original contributions presented in this study are included in the article. Further inquiries can be directed to the corresponding author.
